# Strain-tunable optical microlens arrays with deformable wrinkles for spatially coordinated image projection on a security substrate

**DOI:** 10.1038/s41378-022-00399-7

**Published:** 2022-09-14

**Authors:** In Sik Choi, Seongho Park, Sangheon Jeon, Young Woo Kwon, Rowoon Park, Robert A. Taylor, Kwangseuk Kyhm, Suck Won Hong

**Affiliations:** 1 Department of Cogno-Mechatronics Engineering, Department of Optics and Mechatronics Engineering, Pusan National University, Busan, 46241 Republic of Korea; 2 Research Center for Dielectric and Advanced Matter Physics, Pusan National University, Busan, 46241 Republic of Korea; 3 Department of Physics, University of Oxford, Oxford, OX1 3PU UK; 4 Department of Nano-Fusion Technology, Pusan National University, Busan, 46241 Republic of Korea

**Keywords:** Engineering, Optical materials and structures

## Abstract

As a new concept in materials design, a variety of strategies have been developed to fabricate optical microlens arrays (MLAs) that enable the miniaturization of optical systems on the micro/nanoscale to improve their characteristic performance with unique optical functionality. In this paper, we introduce a cost-effective and facile fabrication process on a large scale up to ~15 inches via sequential lithographic methods to produce thin and deformable hexagonally arranged MLAs consisting of polydimethylsiloxane (PDMS). Simple employment of oxygen plasma treatment on the prestrained MLAs effectively harnessed the spontaneous formation of highly uniform nanowrinkled structures all over the surface of the elastomeric microlenses. With strain-controlled tunability, unexpected optical diffraction patterns were characterized by the interference combination effect of the microlens and deformable nanowrinkles. Consequently, the hierarchically structured MLAs presented here have the potential to produce desirable spatial arrangements, which may provide easily accessible opportunities to realize microlens-based technology by tunable focal lengths for more advanced micro-optical devices and imaging projection elements on unconventional security substrates.

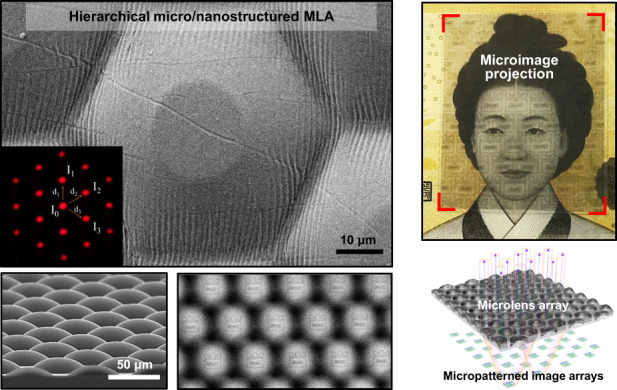

## Introduction

Microlens arrays (MLAs) have garnered significant attention and have been used as an important component in many optical systems due to their broad range of applications^1^, including digital displays^2^, integral imaging^3^, three-dimensional imaging^4^, high-resolution molecular bioimaging^5^, high-density data storage^6^, artificial eyes^7,8^, and optical communications^9^. For example, monolithically integrated MLAs have been beneficial for improving photon collection by planar image sensor arrays in complementary metal-oxide-semiconductor (CMOS) and charge-coupled device (CCD) systems^10^. Moreover, the integration of MLAs for use in enhanced light outcoupling efficiency or homogeneous illumination in light-emitting devices has enabled promising strategies for implementing microscale light sources such as flat panel displays or biomedical devices^11,12^. An integrated MLA with a high fill factor can be considered a 2D optical element that increases light utilization, while multilayer MLAs in the direction of light propagation may contribute to 3D integrated micro/nano-optical systems, which can potentially be applied to laser homogenization, holographic projection processes or other optical inspections^13^. Recently, with the development of optoelectronic engineering and the growing interest in new functional and progressive 3D MLA-based components^14^, nature-inspired optical system design and materials have become a new research topic in this community. In particular, among the various optical materials in biomimetics, compound eyes, found in insects or crustaceans, have been attracting attention as crucial micro-optical elements for optoelectronic mechanical applications^15,16^.

As a new concept in materials design, a variety of strategies have been introduced and rapidly developed to fabricate MLAs that enable the miniaturization of optical systems and improve the characteristic performance in optical functionality^12^. A typical fabrication process involves photolithography or ink-jet printing methods with easy access^17^, and improved techniques have been readily adopted along with the development of other manufacturing tools such as grayscale lithography, laser direct writing, and ion milling. In addition, recent developments in surface tension-driven colloidal particle assemblies also enable the creation of large-scale templates for MLAs as an alternative approach^18,19^; however, a thin layer of spontaneously organized macroscopic building blocks with uniform thickness is required^20–23^. Although these demonstrations have shown intriguing ways to produce customized MLAs while guaranteeing the integrity of optical properties, among these, the most easily accessible method to date is conventional photolithography to adjust the uniform profile of ultraviolet (UV) light distribution; classified studies have explored 3D microlens patterns with optimized photoresist conditions through a single exposure step to create nanostructures-on-microlens^24^. In particular, this method can be most attractive for micro-optical components, considering the reasonable manufacturing cost while maintaining a specific focal length^25^. However, one of the major challenges in the generation of MLAs is reproducibility and assembly accuracy on a large scale.

Here, we present a high-throughput, scalable, and cost-effective approach to effectively generate hexagonally configured MLAs with a focal length ranging from 80 to 120 μm via simple photolithography, optimized electroforming, and a microimprinting process. Unlike general rigid microlenses, the newly developed MLAs consist of elastomeric polydimethylsiloxane (PDMS) that enables them to take a sufficiently thin and deformable form and to fully demonstrate transparent microimaging projection in a flexible format, exploring the possibility of micro-optical security applications^26^. Moreover, simple exposure to a plasma treatment on the prestretched PDMS-MLAs can spontaneously harness the formation of nanoscale wrinkles all over the surface of the microlenses^27^. The formation of nanowrinkles on MLAs is a direct consequence of stress due to the physical confinement effect on a SiO_2_ (i.e., silica) surface produced during oxidation of the PDMS surface by O_2_ plasma treatment^28–30^. In addition, the chosen material (i.e., PDMS) is easily tunable with stress-responsive sensitivity by controlling the nanowrinkles on the MLA, which are sufficiently transparent in microlens applications. As a result, hierarchically micro-nanostructured MLAs can be created within tunable ranges in a precisely controllable manner when they are mounted on a translational stage. Surprisingly, by the interference combination effect of the microlens and nanostructured wrinkles, unpredictable optical diffraction patterns can be characterized by laser irradiation (*λ* = 633 nm) and UV–vis measurements. This unique micro-optical benefit was facilitated by the interference combination effect of the elastomeric MLA and strain-tunable nanowrinkles. While most results on the optical properties of nanowrinkled PDMS were obtained by using flat surface geometries with an elongated or shrunken state, as previously reported, our study showed the integration of a microlens covered with nanowrinkles to take advantage of a tunable focal length capability. Moreover, the additional optical performance of the as-fabricated elastomeric MLAs was also demonstrated by testing their image projection capabilities. Clearly, the state-of-the-art technology and unique structures presented here are appealing because of their ability to engineer the nanowrinkle formation with a simple process to enable a new class of multifunctional optical components that may be applicable to 3D holographic imaging, Shack–Hartmann wavefront sensors, and deformable displays as finely controlled diffusers in the near future^31,32^.

## Results and discussion

### Fabrication of elastomeric MLAs

**Fig. 1:**
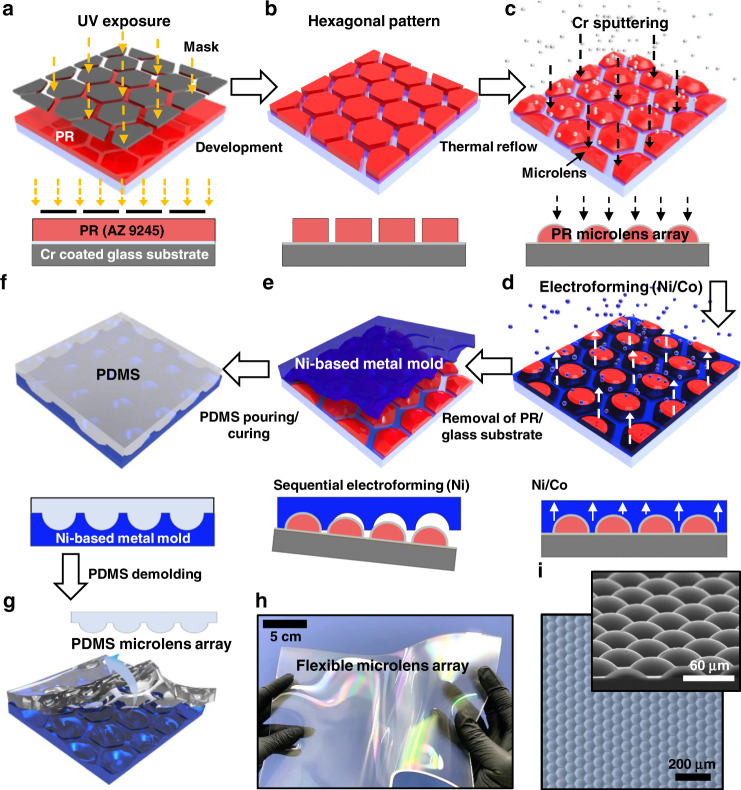
**Schematic description of the fabrication sequence for MLAs**. **a**, **b** Hexagonally arranged PR arrays formed by photolithography. **c** Converting PR arrays to hemispherical microlenses through a thermal reflowing process and subsequent Cr layer deposition on the PR surface by sputtering. **d** Electroforming process with a Ni/Co alloy for metal mold preparation on hemispherical PR arrays and the removal of photoresist to separate the Ni mold. **e–g** Replication of MLAs by casting and curing PDMS on a Ni mold. **h** Digital image of an elastomeric MLA film on a large scale. **i** Top and perspective SEM image (inset) of the produced MLA

Figure 1 provides a schematic illustration of the sequential processes used to fabricate the elastomeric MLA. In this experimental scheme for a high filling close-packed MLA, we designed a hexagonally arranged hemispherical geometry produced by standard photolithography and a conventional thermal reflow process. The first step involves spin casting the positive tone photoresist (PR, AZ 9245) at 1500 rpm for 30 sec, yielding an optimized thickness (8 μm) on a Cr (100 nm)-coated glass substrate. Subsequently, after baking for 10 min at 110 °C, a UV light source (*λ* = 365 nm, I-line mask aligner) was used to expose the entire substrate (dosage = 480 mJ cm^−2^), which was fully contacted with a Cr photomask (Fig. 1a), and the development was performed for 10 min (AZ 300MIF), leaving behind isolated hexagonal pillar islands with a sharp contrast that was identical to the designed photomask (Fig. 1b, the exposed gap between the PR: 3 μm). Next, the patterned PR substrate was placed on a hot plate (60 sec at 135 °C) for a typical reflow process and slowly cooled to room temperature. Finally, a hexagonally packed plano-convex MLA consisting of PR (i.e., the master stamp) was successfully fabricated (Fig. 1c), which was explored by a 3D laser scanning microscope (VK-9700K, Keyence), as shown in Fig. S1a. Generally, the integrity of the MLAs resulting from the reflow process can be controlled by the initial thickness of the PR and the configuration of the cylinder structure (e.g., diameter), which determines the sag height and the radius of curvature of a microlens^33^. In fact, in addition to this geometrical factor, the driving force for the formation of hemispherical lenslet arrays by the reflow process is surface tension following surface energy minimization at the elevated glass transition temperature (*T*_g_) of the photopolymer (AZ 9245, ~125 °C)^34^. In addition, the curvature of the microlens, as a result of the optimized reflow process, can be controlled by the initial volume of the cylindrical PR (i.e., height and diameter) formed on a substrate (Fig. S2 and S3). Interestingly, in some cases, a flat central zone in the center of the microlenses was found after the thermal reflow process, compared to the fitted profile for an ideal spherical shape, which is shown in Fig. S1b. In our system, these centrally flat-top MLAs can be intentionally reproduced by the delicate control of the temperature and reflow time (i.e., 60 sec at 135 °C) on the same hexagonal pillars of the PR. As measured, the aspect ratios of each microlens for the flat-top and fully reflowed samples were ~0.18 and ~0.22, respectively. It should be noted that the gap spacing among the microlenses (3 μm) was critically maintained with a small increase in aspect ratio, regardless of the reflow process without notable sliding of isolated PR patterns in all cases. Thus, the packing density yields 1:1 imaging matched with the designed photomask, generating hexagonally arranged spatial orders, which is an important factor in determining the optical characteristics of MLAs.

To easily duplicate the soft material (i.e., PDMS) for MLAs on a large scale, we created a metal replica stamp through an electroforming process directly on the prepared PR patterned Cr/glass substrate. Among the various soft lithography methods, template molding offers highly reproducible replication from the mother substrate. In the following, for this purpose, Cr was sputtered at 17 nm to secure the conductivity of the polymeric master mold with a critical thickness for the Ni-Co electroforming process; the growth of a metal alloy transpired in a nickel sulfate-based solution for ~18 h (Fig. 1d)^35^. This allows for more sophisticated duplication than conventional PR-based soft lithography, overcoming limitations such as unwanted delamination of the master PR mold from the substrate (Fig. 1e)^36,37^. Notably, a serious drawback in producing replica molding for MLA was an intrinsic limitation in the number of replications, mainly due to unwanted chemical interfacial bonding with the PR and prepolymer resin when molding the replicas, including PDMS^38^. As a consequence, thus far, there have been difficulties in the scale-up process of micromolding using PR-based molds. Importantly, we were able to overcome this serious bottleneck with our approach by preparing a Ni-based metal mold that can be used for a semipermanent number of replications. Our developed technology may be beneficial for reliability and expandability by applying other polymeric resins, enabling mass production of microlenses for possible other applications. Indeed, the prepared PDMS prepolymer mixed with the curing agent was poured onto the Ni mold and then crosslinked in an oven for 30 min at 75 °C (Fig. 1f). Finally, the replicated PDMS-based MLA, as large as 15 inches, was gently demolded from the Ni stamp (Fig. 1g, h). The replicated surface was measured by scanning electron microscopy (SEM), which clearly displays hexagonally close-packed MLAs (i.e., ~168,000 inch^−2^), ensuring critically ordered regularity with high integrity, as shown in Fig. 1i and S4.

### Microimage projection as a security substrate using elastomeric MLA

**Fig. 2:**
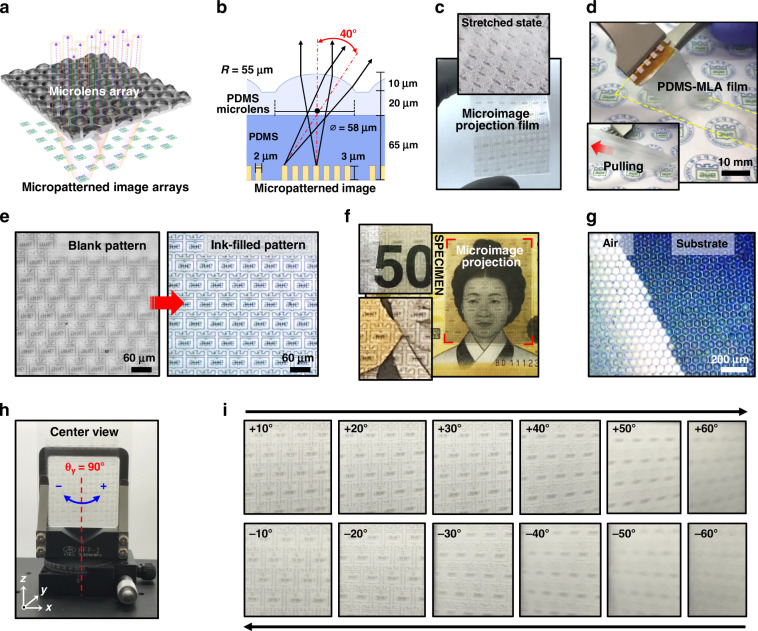
Optical characteristics of the microimage projection film as a security substrate using elastomeric MLA. **a** Conceptual schematic illustration of a layered microimage projection film using MLA and MPIAs. **b** Side-view schematic of the unit cell dimensions and the details of the integrated elastomeric security film substrate with underlying high-resolution MPIA. **c** Representative digital image of a flexible security film (4 × 5 cm) and stretchable form with large image distortion. **d** Digital images of the deformable security film, consisting of the background MPIA and MLA, on a transparent carrier substrate. **e** Magnified optical micrographs of the MPIAs consisting of PDMS; as-prepared sample (left) and improved security features of the ink-filled pattern in black/white (right). **f** The PDMS-based security film transferred onto the surface of the banknotes from the Bank of Korea located on the portrait region marked with red brackets; the security symbols of arrays were clearly projected through the transparent PDMS. **g** Optical micrographs of conformal contact demonstration of the elastomeric PDMS microimage projection film on a substrate; each dark region and bright region represent conformally contacted and floating areas, respectively. **h** The freestanding microimage projection film placed in the optical sample holder. **i** A series of digital images rotated up to ±60 degrees from the perpendicular viewing angles for the visual characteristics

Figure 2a schematically illustrates a stacked configuration of each high-density microlens and underlying finely patterned microscopic image arrays. To evaluate the optical effect of the security-grade newly developed MLA, we designed a unique laminated film consisting of the same or different types of elastomers (i.e., PDMS/PDMS or PDMS/thermoplastic polyurethane, TPU); in some cases, the lamination of TPU (i.e., Young’s modulus ~20 MPa) improves the mechanical resistance compared to the use of PDMS/PDMS (i.e., Young’s modulus ~2 MPa). Sheets of micropatterned image arrays (MPIAs), prepared separately as an image projection-enabled symbol, can be conformally contacted and fully bonded to the planar bottom surface of the MLA. To produce the MPIAs, we used the same photolithography and molding processes, as described in Fig. S5. In the laminated form, the phenomenon of Moiré magnification occurs when the MLA is aligned and overlaid parallel to the symmetric axis of the MPIA with a similar periodicity (mismatch allowed within 1° based on a parallel line)^39^. Thus, a periodic arrangement of highly magnified macroscale patterns can be observed as identical objects; the patterns were enlarged so that they could be visible to the naked eye. Figure 2b provides a side-view schematic of the unit cell dimensions and the details of the integrated elastomeric security film. For effective floating imaging, the bottom layer was defined to be 65 μm thick, including the structure of the MPIA with a 2 μm line width and 3 μm height (a single size of symbol = 60 μm). The height of the top MLA layer was fixed at 30 μm with a radius curvature (*R*) of ~55 μm. Notably, the height of the top layer of the microlens array was fixed at 30 μm with a radius curvature of ~55 μm. The distance between the center of the MLA and MPIA and micropatterned images was spatially arranged to be exactly ~95 μm for clear image projection. Since the focal length is proportional to the radius of curvature, the thickness of the MLA layer is sensitive and affects the fields of the focal plane to be projected in the far-field. In this experiment, our method provides the necessary MPIA resolution (i.e., grayscale university logo as an example image) and excellent control over MLA geometries in a range of focal lengths of ~100 μm. The height of all the films could be easily controlled by the amount of PDMS or TPU poured on each mold, and then the prepared sheets were precisely aligned for the overlay registration under an optical microscope. In the next step, the physically contacted multilayer structures were cured in the oven to form a thick single-layered film. Figure 2c shows a representative digital image of a flexible microimage projection film (4 × 5 cm) and stretchable form, with very large image distortion observed when stretched (inset). This elastomeric microimage projection film is mechanically robust but difficult to handle because of its exceptionally low bending stiffness. Thus, practical manipulation requires some type of supporting substrate in the process; thus, a transparent plastic film as a carrier substrate (e.g., polyethylene terephthalate, PET) was facilitated, as demonstrated in Fig. 2d and Movie S1 (Supplementary Information). In the following, the blank micropatterned surface-relief areas (background MPIA, yellow bottom patterns in Fig. 2b) were filled with a single type of ink in black to enhance visibility. Figure 2e shows enlarged optical micrographs of the MPIAs, indicating the blank pattern of the as-prepared sample (left) and the ink-filled pattern of the clearly improved security features in black/white (right) via a micro gravure-like technique. By retaining attractive visual effects with the capability of withstanding extreme conditions (e.g., folding, bending, and stretching), these thin elastomeric security films are suitable for the requirements of realistic potential applications such as security labels attached to banknotes, credit cards, valuable documents, or other government-issued identification cards^40,41^. As a result, the optical performance of the security film was demonstrated, as shown in Fig. 2f. In this test, the PDMS-based security patch was transferred to the surface of currently used banknotes from the Bank of Korea (see the portrait region marked with a red bracket), and the security symbols of arrays were clearly projected due to the transparent characteristics of PDMS (inset: the single symbol size was ~5000 μm, with a magnification of more than 80 times from the MPIA). Our original intention lies in the demonstration of a security film that can be used for banknotes or valuable documents by facilitating a mechanically robust PDMS microlens with the capability of withstanding extreme conditions (e.g., folding, bending, and stretching). Because the thickness level was found to be sensitive and critically affected the fields of the focal plane to be projected in the far-field, identified by the naked eye, the optical quality of our PDMS-MLA was clearly verified in this application. In this demonstration, the predominant integrity of the curvature of the microlens yielded a perfectly matched focal length to be used for security films when viewing the magnified MPIA with a clearly discernible projection over the MLA. Moreover, a further aspect of the excellent tackiness of PDMS operating in a transparent film enabled conformal contact onto the surface of banknotes, as observed in Fig. 2g^42^. This implies that the simple application of an additional adhesive film (i.e., optically clear adhesive, OCA film) may allow for easy lamination onto other surfaces, such as paper, fabric, polymer sheets, or even curved arbitrary substrates^43^. The thickness of the security film was thinner than ~100 µm, which may expand its applications in fields sensitive to layer thickness. Furthermore, due to the nature of image projection, small-scale moving symbols can be displayed when the sample is rotated in any direction. For this observation, as shown in Fig. 2h, the freestanding image projection film was placed in an optical sample holder, and the sample was rotated up to ±60 degrees from perpendicular viewing angles. Figure 2i represents a series of the measured results for the visual effect. Apparently fine symbol resolution, reaching ±40 degrees without distortion of the images, was detected macroscopically, converting the distance from the reference point to ~1 cm or more (Fig. S6). This is presumably due to the lithographically defined MPIAs with high resolution and a perfectly matched alignment of the MLA on the underlying image arrays. We envision that easy access to materials at such scales may accelerate the adoption of related lithographical technologies combined with nanopixel generation or functional inks, and attractive visual effects can lead to the development of micro-optic security systems with specific MLAs for advanced holographic image presentation^44^.

### Formation of nanowrinkled structures on the MLA surface

**Fig. 3:**
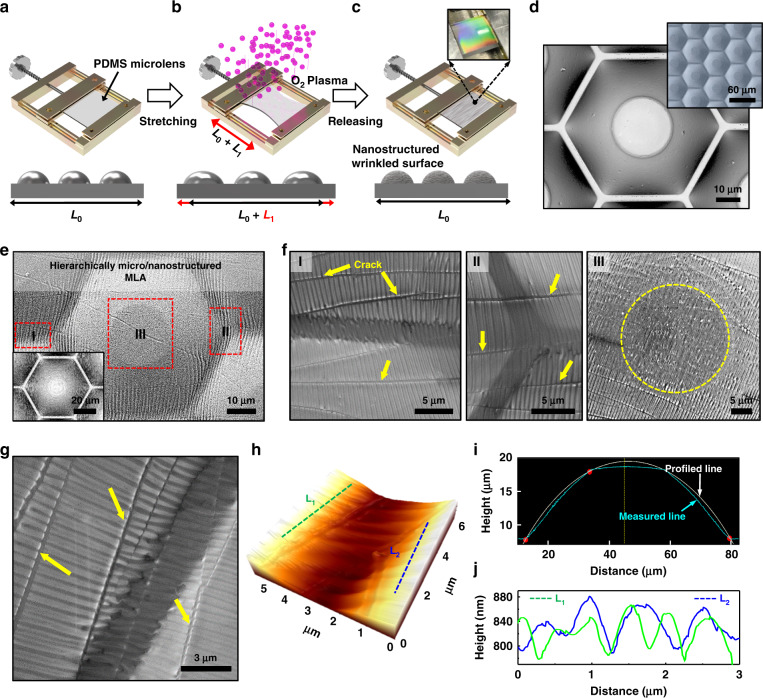
Hierarchical nanostructured wrinkles on the PDMS-based microlens surface. **a–c** A series of schematic illustrations of nanowrinkle formation by prestrain, oxygen plasma treatment in the stretched state, and release to the initial state. **d** 3D laser scanning image and SEM image (inset) of the MLA without O_2_ plasma treatment. **e** Macroscopic SEM image of the MLA surface with nanostructured wrinkles over the entire area. **f–g** Magnified wrinkled nanostructures; yellow arrows indicate naturally formed cracks. **h** AFM measurement of the nanowrinkled surface on the MLA. **i** 3D laser scanning profile. **j** AFM height profiles corresponding to the dotted lines of L_1_ and L_2_ in **h**

In addition to the attractive security features of the microimage projection described above, we propose a facile strategy to modify the microlens surface with deformable nanostructures (i.e., wrinkles), utilizing elastomeric MLAs as multifunctional optical films. Figure 3a–c schematically depicts the surface modification process for fabricating highly aligned nanowrinkles on the PDMS-based MLAs. First, a cut MLA sheet was firmly mounted at both ends of a customized translation stage and mechanically prestrained in the transverse direction for optimized elongation (*L*_1_). Next, the surface of a siloxane-containing polymer (i.e., PDMS) was uniformly oxidized by O_2_ plasma treatment to produce a very thin SiO_2_ layer (~6–12 nm) on the exposed MLA surface. In the following, when released to its initial state (*L*_0_), nanostructured wrinkles were formed spontaneously, perpendicular to the elongated direction by the strain-dependent confinement effect between the brittle SiO_2_ layer and the soft PDMS (inset in Fig. 3c), as reported in previous studies (Fig. S7)^27–29,45,46^. Recently, we have demonstrated biologically inspired MLAs composed of hierarchical nano-on-microstructures using sequential unconventional lithographic methods that involve more or less cumbersome multiple steps in the process^47^. However, the experimental concept in this study is more advantageous for producing similar hierarchical structures in a simple and cost-effective manner. Figure 3d presents the initially prepared hexagonally configured microlens surface with a perfectly smooth surface, in which the surface profile and the zoom-in surface image were measured by 3D laser scanning microscopy and SEM (inset), respectively; the detailed microlens configuration is presented in Fig. S8. Upon releasing the strain from the oxidized PDMS after O_2_ plasma treatment, highly ordered wrinkled nanostructures were built on the symmetric flat-top MLA by delicate tuning of the prestrain value from 10–20% under a controlled oxidation duration of up to 3 min. A typical SEM image appeared (Fig. 3e) under the representative condition (1 min oxidation, 10% prestrain); a tremendous amount of the nanotextured wrinkles with submicron periodicity were uniformly created on the surface of the MLAs over the entire surface area, mimicking a biomimetic cuticular appearance such as firefly light organs^48,49^. As reported previously^50^, these nanostructured wrinkles are naturally generated by the gradient difference of Young’s modulus between the brittleness of SiO_2_ in the PDMS matrix. To examine the nanowrinkled structure on the MLA, we measured the surface in detail at the selected major points placed on the top, valley, and ridges, indicated by the boxed regions in Fig. 3e. As displayed by the magnified images in Fig. 3f, the nanostructured sinusoidal wrinkles fully cover the protruded microlenses and narrow ridges between microlenses with some randomly embedded cracks (yellow arrows), including the central flat zone of the microlens (right panel in Fig. 3f marked circle: the center of the microlens). This observation clearly confirmed a highly uniform density of continuously formed wrinkles that is comparable to those of other regions in terms of the defined wavelength scale. From the marked region in Fig. 3g, the surface profiles measured by atomic force microscopy (AFM) reveal the nanotextured wrinkles in detail (Fig. 3h). Importantly, the 3D laser scanned micrograph indicates that the original lens geometry has been fully recovered after the releasing step (Fig. 3i). The AFM height profile also shows that the nanostructured wrinkles were formed in a relatively regular fashion in wavelength (*λ*) and amplitude (*A*); the average amplitude of nanowrinkles (i.e., height) for each region is ~80 nm, and the wavelength distribution ranges from 700 to 850 nm (Fig. 3j). The calculated structural analysis of the periodic wrinkles and the detailed correlation with the amplitude are summarized in Fig. S9 for more information^51,52^.

### Optical performance of the MLA structured with nanowrinkles

**Fig. 4:**
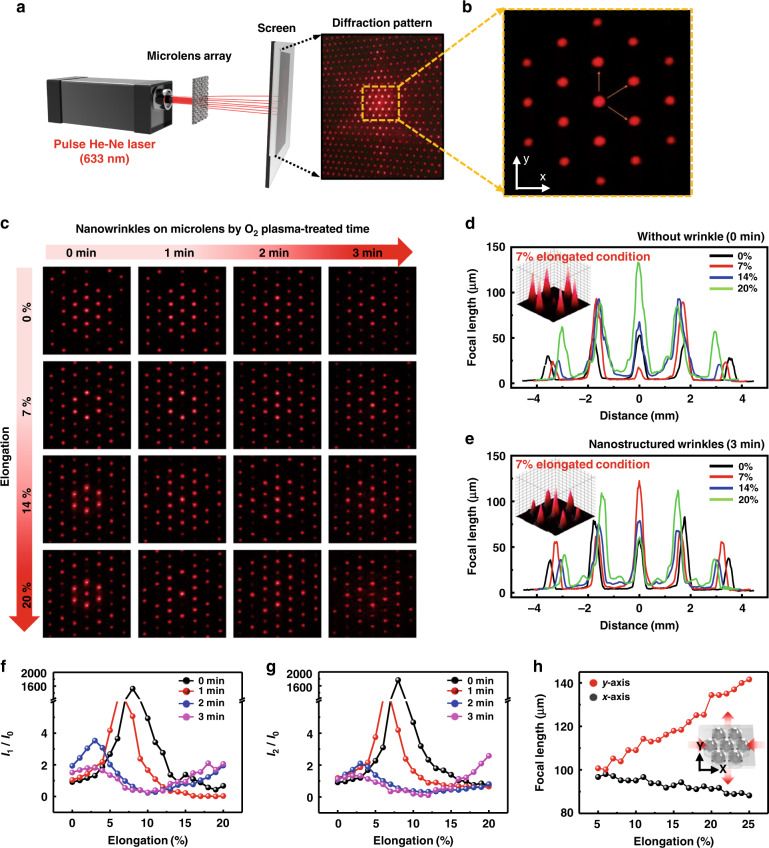
Optical performance of the MLAs with nanowrinkles. **a** The experimental setup for optical characterization using MLAs. **b** Center part of the far-field diffraction pattern denoted from the 0th-order diffraction spot to the 1st-order diffraction spots **c** A collective set of diffraction patterns generated by subjecting the MLAs to incident laser irradiation under the different surface oxidation and elongation conditions. **d**, **e** *y*-axis orientation intensity profile as a function of distance for the different elongation states without and with nanostructured wrinkles, respectively. **f**, **g** Integrated intensity ratio (*I*_n_/*I*_0_) as a function of the elongation for the different oxidation times. **h** Characteristic changes in the focal length of the *x-* and *y*-axes under uniaxial elongation

To evaluate the optical performance of the MLA structured with nanowrinkles (N-MLAs), we used a laser setup, as shown schematically in Fig. 4a. A far-field diffraction pattern was obtained by transmitting the light source (He-Ne laser, 633 nm) directly through the prepared MLA. Without stretching, the typical diffraction pattern is dominated by the periodic spatial arrangement of the hexagonal shape of the MLA. Unlike a conventional rigid MLA, the PDMS-based elastomeric MLA can be easily deformed by an external field (i.e., tensile strain) with fine controllability. The tunable PDMS-MLA can be considered a new technology by changing the strain without permanent deformation, which can also be recovered into the original shape when released^53,54^. In particular, the elongation of the MLA along a uniaxial direction leads to a tunable focal length. In each condition, the curvature of the individual microlens changes^55^, and the reconstruction of nanowrinkles formed on the MLA surface changes the diffraction patterns^56^. The external strain along the uniaxial direction was carefully engaged using a motorized translation stage. We observed a significant change in the intensity distribution of the diffraction patterns. For the quantitative analysis of far-field diffraction images, the intensity of the diffraction spot parallel to the elongation direction was denoted by *I*_1_, and the diffraction points located in the direction perpendicular to the extension direction were set to *I*_2_ and *I*_3_. The intensity ratio of each diffraction spot and the intensity of the laser beam spot located in the center (set as *I*_0_) were compared by elongating the sample (Fig. 4b). Figure 4c shows a set of diffraction patterns of each for increasing elongation up to 20%, where the samples are O_2_ plasma-treated N-MLAs under different conditions. It is noticeable that the plasma exposure time obviously affected the optical properties of the N-MLA. For example, the top image in the first column of Fig. 4c shows a diffraction pattern from the as-prepared MLA without elongation and O_2_ plasma treatment, where the intensity of 0th-order diffraction is dominant over other diffraction spots, as plotted in Fig. 4d. By changing the elongation, the intensities of the higher-order diffraction spots became enhanced, and the diffraction contrast of the spot patterns was remarkably distinguishable within the range of stretching steps (Fig. 4d, e). More importantly, we found that the diffraction patterns are also highly dependent on the O_2_ plasma treatment (i.e., the nanowrinkle effect formed on the MLA surface). Based on this background, the *I*_n_/*I*_0_ values as an elongation change are summarized in Fig. 4f, g.

Figure 4f shows the elongation dependence of *I*_1_/*I*_0_ along the parallel direction (i.e., *y*-axis). All the samples appeared to have a similar contrast (*I*_N_/*I*_0_ ≈ 1–2) in the initial state. However, as the samples were stretched, the ratio of each diffraction point was observed to have two types, which depend on the O_2_ plasma treatment time. As shown in Fig. 4c, d, in the case of an O_2_ plasma treatment time of 0 min or 1 min, the intensity of the center spot approaches 0 when the samples are elongated by 7–8%. In contrast, in the same elongation state, the intensity of the center spot increases when the O_2_ plasma treatment time is 2 or 3 min. This suggests that the possible reconstruction of wrinkled nanostructures by elongation tunes the sensitivity of the microlens under critical conditions^56^. In detail, elongating the samples by ~7–8% for 2 min and 3 min will result in intensities at the central spot and the surrounding spots that are almost identical (*I*_n_/*I*_0_ is close to 1). This result suggests the possibility of creating more efficient diffractive optical elements for light diffusers. The difference only occurs when the sample is elongated by less than 15%. If the sample was extended by more than 15%, then similar results were observed regardless of O_2_ plasma treatment. Presumably, the nanowrinkles were restored to the less corrugated state (i.e., smooth surface) and became very similar to that of the sample without plasma treatment. These results seem to indicate that flattening may occur as the nanowrinkles spread across the surface, as reported previously^56^. Moreover, it is worth noting that the organized structure exhibits a similar effect to a transmission diffraction grating because the nanowrinkles on the PDMS surface by the optimized O_2_ plasma can be created perpendicular to the direction along which the prestrain is applied (Fig. 3a). Typical diffraction gratings are optical elements consisting of hundreds to thousands of grooves per mm, and they spatially separate light into constituent wavelength bands. However, unlike general diffraction gratings, elastomeric N-MLA has the potential for a novel diffractive optical device because the characteristic periodic wavy grooves (i.e., highly aligned nanowrinkles) can be transformable by simply tuning the strain fields^57^.

The focal length (*f*) of ae plano-convex lens can be estimated using *f* = (*r*^2^ + *h*^2^)/2 *h*(*n* − 1) − *h*(*n* − 1), where *r*, *h*, and *n* are the radius of the lens, the height of the lens, and the refractive index of PDMS, respectively^58^. In fact, the focal length (*f* ≈ 100 μm) along the *z*-axis can be obtained because the size of the MLA (⌀ = 58 μm) is isotropic, which is consistent with the optical simulation conducted by the finite-difference time-domain method (FDTD, Fig. S10). On the other hand, if the height of the lens is shortened by elongation (i.e., stretched state), then the focal length increases as the radius of the lens curvature changes. Successive changes in focal length directed by elongating along the *x*- and *y*-axes naturally induced a spherical aberration. For example, as shown in Fig. 4h, the radius of the microlens, *r*, was measured to be ~29 μm in the fixed state without elongation. However, if the sample is stretched by 20% along the *y*-axis, then *r* increases to ~33 μm (*f* ≈ 142 μm). In the case of the *x*-axis, *r* decreases from ~29 to ~26 μm, resulting in a focal length of ~91 μm (Fig. S11). Consequently, even if a strain is engaged along the *y*-axis, the compressive forces on the microlenses are simultaneously applied to the *x*-axis, which is perpendicular to the direction of the applied field; the somewhat delicate deformation of the nanowrinkles is possibly nonuniform, which may lead to additional optical effects, as described earlier^56^. These conditions resulted in an unexpected physical response, as shown in Fig. 4d, e. Interesting results can be obtained when viewing remotely positioned images using deformable nanowrinkles formed on MLAs. This result is expected to promote a recovery effect associated with the optical aberration capabilities.

**Fig. 5:**
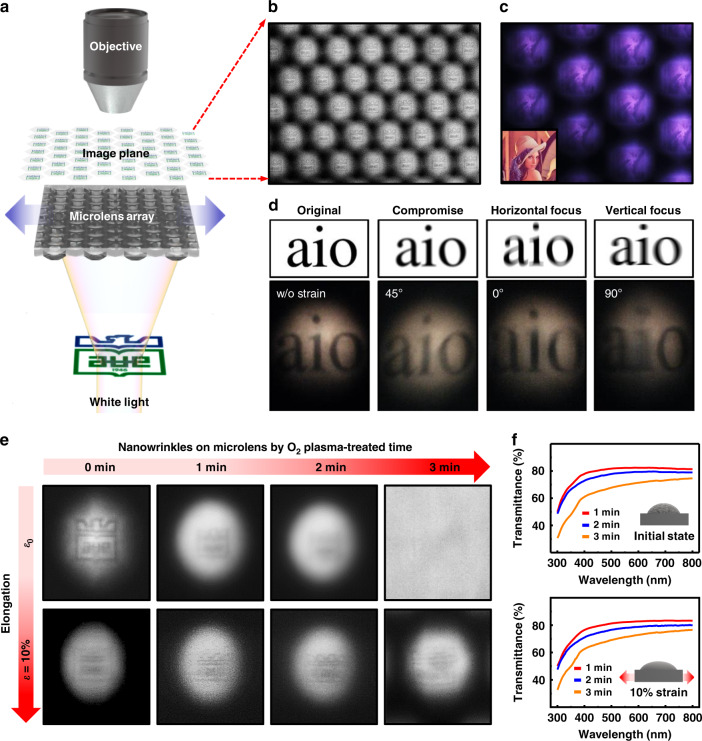
Focused imaging test for the MLAs and N-MLAs. **a** Schematic configuration of the experimental setup consisting of a CCD camera, an objective lens, and a white light source that was equipped under the example images. **b**, **c** The imaging performance of the as-prepared MLA in which the university symbol and Lenna image appeared as focused objects. **d** The virtual astigmatism test through the stretched MLA rotating at a certain angle (θ = 45, 0, and 90°) on the standard reference text (upper row) compared with the focused imaging results (bottom row). **e** A set of focused images for tunable N-MLAs under different oxidation and uniaxial 10% tensile strain conditions. **f** UV–Vis transmission spectra for the unstretched (upper) and strain-engaged cases (lower) within the wavelength range from 300 to 800 nm for the N-MLAs at different oxidation times

Based on the above result, unusual optical properties due to the coupling interference by mechanical deformation and nanostructured wrinkles may lead to dramatic changes in performance if used as monolithic optical modules, which have great potential to expand our work into another viable application^59^. In this study, we performed an imaging test by projecting virtual target images onto the MLAs. Figure 5a shows a schematic configuration of the experimental setup. Here, example images printed on paper (university symbol, Lenna image, or text; 2.5 × 2.5 cm) were placed under the MLAs and illuminated by a white light source. The focused spot images on the microlenses were captured by a CCD camera. As test cases, Fig. 5b,c presents a very clear focused image array (each ×50 and ×100 magnification), displaying excellent imaging performance of the MLA without manipulation or surface modification (Fig. S12). However, a somewhat interesting optical phenomenon was found when a specific tensile strain (i.e., 5%) was applied uniaxially to the MLA. As shown in Fig. 5d, the commonly used standard reference (i.e., “aio” text) was tested for clear vision through the stretched MLA rotating at a certain angle (*θ*), and apparent results, similar to those seen in patients with regular astigmatism, were obtained^60^. To clarify the general condition under a microscopic environment, a standard test was also performed using a cross-bar image with or without a tensile strain field, as presented in Fig. S13. This outcome is presumably due to the slightly different focal length in the *x*- and *y*-axes, which creates a distorted and blurred field of view through the MLA by a difference in the degree of refraction curvature (i.e., the same manner as the irregular cornea). Because astigmatism refers to the symptoms of blurred vision when light rays on the eye fail to focus at a point on the retina by the multiple focal points from the difference between the vertical and horizontal focal lengths, a similar condition could be provided by the deformable MLA. As described earlier, when the MLA is deformed in a uniaxial direction, the individual microlenses are stretched in the tensile direction and compressed in the lateral direction, thereby resulting in intersecting beam waists, as shown in Fig. 4. Because regular astigmatism is caused by an irregular curvature of the cornea that can deviate the focal point of light, this optical measurement validates that our mechanically tuned MLA is transformed into a shape similar to that of a toric lens with small feature sizes. To correct or generate astigmatism, a toric lens is widely used with different focal lengths in two orthogonal axes that work as a combination of spherical and cylindrical lenses^61^. This implies that our optical platform presented here can be an astigmatism-tunable microlens simply by employing delicate control of the elastic material (i.e., PDMS) because conventional lens designs do not adequately address the astigmatic optical performance of lenses with tunable focal lengths^60^. Thus, this approach may be applicable for measuring the degree of astigmatism by a simulation of an emmetrope’s view via specific projected images or to minimize the astigmatism effects practically used in a head-mounted display with finely designed MLAs^62^.

In addition, a noticeable optical characteristic was observed in the N-MLA samples by adequate control of the surface oxidation and an appropriate field of strain (i.e., 10% elongation). The scattering effect of nanowrinkles clearly made the visualized images more blurred as the oxidation time increased (Fig. 5e). In particular, the nanowrinkles on the MLA produced by relatively long oxidation (i.e., 3 min) efficiently scattered most of the light and found that the focused image was not measured and invisible, as shown in the rightmost panels of Fig. 5e. It is worth noting that the delicate control of the strain field over the N-MLAs naturally reduced the scattering effect mainly due to the well-known recovery of the reversible nanowrinkles to the semismooth “silica skin” state initially formed^27,51^. Therefore, a smoother surface profile of the microlens by flattening with fewer defects minimized the scattering effect from the nanowrinkles, suggesting a better visualization of images upon mechanical stretching compared to the case of unstretched MLA (Fig. S11c). Because the focused imaging process using the MLA and N-MLA is transient, the prediction and direct measurement of the flattened surface profiles depending on the deformation is difficult, especially for our complex surface profiles. However, a relevant aspect of transmittance for the regime of the *I*_0_th spot was to substantiate that the stretched state may change the strain-induced surface topography of nanowrinkles over the visible range, as illustrated by the series of UV–Vis measurements in Fig. 5f. Compared to the UV–Vis data on the originally provided MLA, the strain field simultaneously changed the focal length and transmittance, as presented in Fig. S11. In addition, the synergistic effect of the unfolded nanowrinkles on the N-MLA clearly contributed to the optical penetration of light with enhanced transmittance. The above set of experiments reveals that the transmittance is increased by ~5% in the 633 nm wavelength range. These permeability changes in the N-MLA are expected to be readily used for highly integrated security substrates because our N-MLA can generate multicoordinated overlay images on the microlens by slightly tuning the focal length in the vertical direction of the *y*-axis (i.e., elongation) and the *x*-axis (i.e., compression), as demonstrated in Fig. S14 and Movie S2 (Supporting Information). Notably, as seen in the demonstration of the microimage projection, the transmittance effect was minimized to magnify the underlying MPIA with a well-matched focal length with a clear view field within ±30 degrees. In contrast, the focused imaging from the N-MLA with the presented microlens design is limited to a highly transparent micro-optical element for optoelectronic mechanical applications. In this regard, a systematic study of multiprojection image transitions in equiaxial deformable MLAs is currently underway with a newly developed design set of MLAs.

## Conclusion

In summary, we developed a simple yet robust lithographic technique for creating highly ordered arrays of microlenses over a large area (i.e., 15 inch scale) and hierarchically structured nanowrinkles on the surface of the microlens via a simple process of O_2_ plasma treatment on prestrained elastomeric MLAs. This platform of optical materials, which consists of fully covered nanostructured wrinkles on the MLAs, can serve as a unique optical element producing unexpected optical diffraction patterns by the combined effect of the optical interference^63^. In addition, the holographic optical performance of the elastomeric MLAs was also demonstrated by testing their imaging capabilities for micro-optical security applications. The resulting collective set of optical characteristics presents a new class of multifunctional physically flexible and stretchable films that can be implemented in other optoelectronic devices or spatially coordinated image projection by facilitating a tunable interface to deliver unprecedented optical signals. We envision that the ability to replicate hierarchically “micro-nano” structured MLAs and to position them into a desired spatial arrangement may provide opportunities to realize the potential of microlens-based technology by tunable focal lengths for more advanced micro-optical devices and imaging elements on unconventional security substrates^7,16,47,64^.

## Materials and methods

### Preparation of microlens arrays

For the fabrication of a hexagonally close-packed MLA, standard photolithography was used. The PR film (AZ 9245, thickness: 8 μm) was prepared by spin coating at 1500 rpm for 30 sec on a 100 nm-thick Cr-coated glass substrate. The hexagonal pillar islands were patterned after development. A conventional thermal reflow process was used to transform the hexagonal pillars into hemispherical MLAs by applying heat at 135 °C for 60 s via a hot plate; the radius of curvature for the MLA was controlled by the initial thickness of the PR. To replicate the PR-based microlens structure, Cr (17 nm) was deposited on the surface of the microlens using an RF sputtering system, and Ni was then deposited by a controlled electroforming process in an electrolytic solution for 18 h to produce a metal mold with a thickness of 500 μm. Next, PDMS (Sylgard 184, Dow Corning, Midland, MI) was prepared with a standard mixing ratio of 10:1 (prepolymer to the crosslinking agent) and poured into the Ni-based MLA mold; microbubbles were completely removed by applying a vacuum in a desiccator before curing at 75 °C for 1 h. After crosslinking, a PDMS-MLA film formed on the metal mold was gently peeled off. A DC plasma system was used for wrinkle formation on the MLA surface with the following conditions: O_2_ gas flow rate of 100 sccm, power of 100 W, and pressure at 5 × 10^−5^ bar.

### Structural characterization

The wrinkled structure of the microlens surface was measured via an optical microscope (Olympus, BX51, Tokyo, Japan) and a field emission scanning microscope (Carl Zeiss AG-SUPRA 40VP, 5–10 Kv, Germany)). The shape analysis and the radius of curvature of the corrugated structure were measured using a laser (Keyence Corp., Osaka, Japan) and AFM (XE-100, Park systems Corp, Suwon, Korea).

### Optical characterization

For optical characterization, we used a He-Ne laser (*λ* = 633 nm, Thorlabs, HNLS008L-EC, Newton, MA, USA). The incident angle of the laser was normal to the MLAs mounted on a motorized translation stage to stretch them in a uniaxial direction. The diffraction pattern was measured using a conventional digital camera under the same conditions (ISO, shutter speed and aperture) at a distance of 300 mm. The measured data were integrated into the intensity of each spot using the ImageJ program, which was used to calculate the set of ratio values of the integrated intensity between 0th^-^ and 1st-order diffraction spots. An optical microscope was used to observe a remotely positioned image. The collected images were captured by a CCD camera after passing through ×20 and ×50 magnification objective lenses (Pax-it, PAXCAM2, Mis Inc., USA).

## Supplementary information

Supporting Information

Flexible microlens array

Flexible microlens array
