# Supplementary material for: Strain-tunable optical microlens arrays with deformable wrinkles for spatially coordinated image projection on a security substrate
Source: Microsyst Nanoeng. 2022 Sep 14;8:98. doi: 10.1038/s41378-022-00399-7 (PMC9474807; doi:10.1038/s41378-022-00399-7)
Supplement: Supplementary file 1 — Supporting Information [file 41378_2022_399_MOESM1_ESM.doc]

Supporting Information for

Strain Tunable Optical Microlens Arrays with Deformable Wrinkles for Spatially Coordinated Image Projection on Security Substrate

In Sik Choi^1^, Seongho Park^2,4^, Sangheon Jeon^1^, Young Woo Kwon^3^, Rowoon Park^1^, Robert. A. Taylor^4^, Kwangseuk Kyhm^1^,*, and Suck Won Hong^1^, *

^1^Department of Cogno-Mechatronics Engineering, Department of Optics and Mechatronics Engineering, Pusan National University, Busan 46241, Republic of Korea

^2^Research Center for Dielectric and Advanced Matter Physics, Pusan National University, Busan 46241, Republic of Korea

^3^Department of Nano-Fusion Technology, Pusan National University, Busan 46241, Republic of Korea

^4^Department of Physics, University of Oxford, Oxford OX1 3PU, United Kingdom

*Corresponding author. E-mail: kskyhm@pusan.ac.kr, swhong@pusan.ac.kr

**Supplementary Tables and Figures**


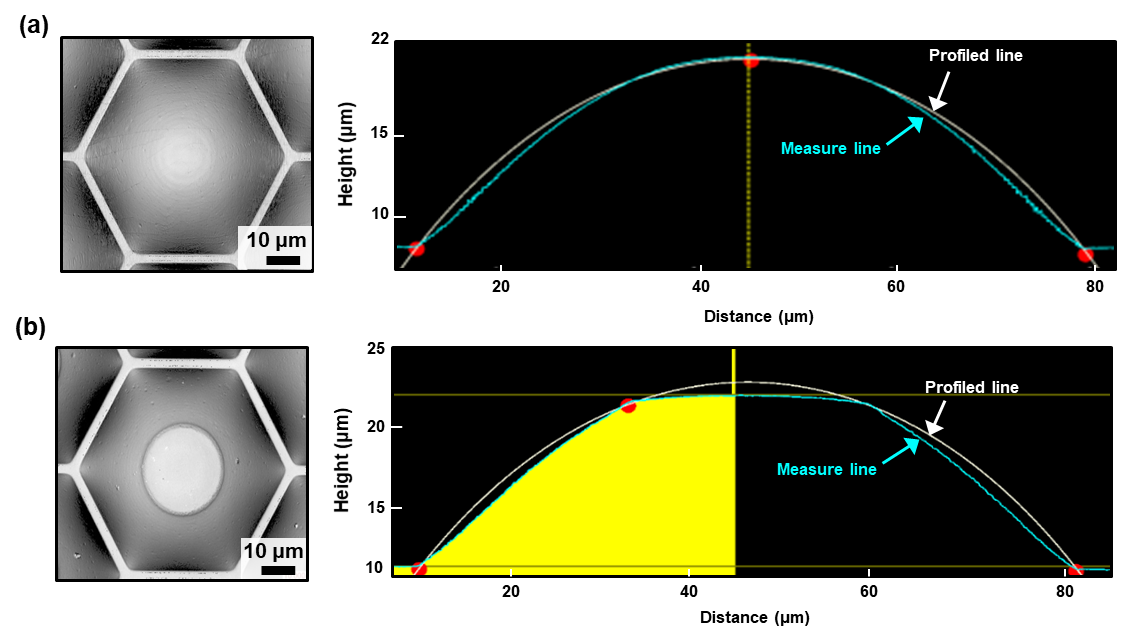


**Fig. S1** Structural analysis of hexagonally configured reflowed PR microlens using 3D laser scanning microscope; the fitted guide profile lines were directly compared to the measured lines. **a** Spherical shaped plano-convex MLA. **b** Central flat-top microlens. Two different type of MLAs were prepared depending on the replica moding process.


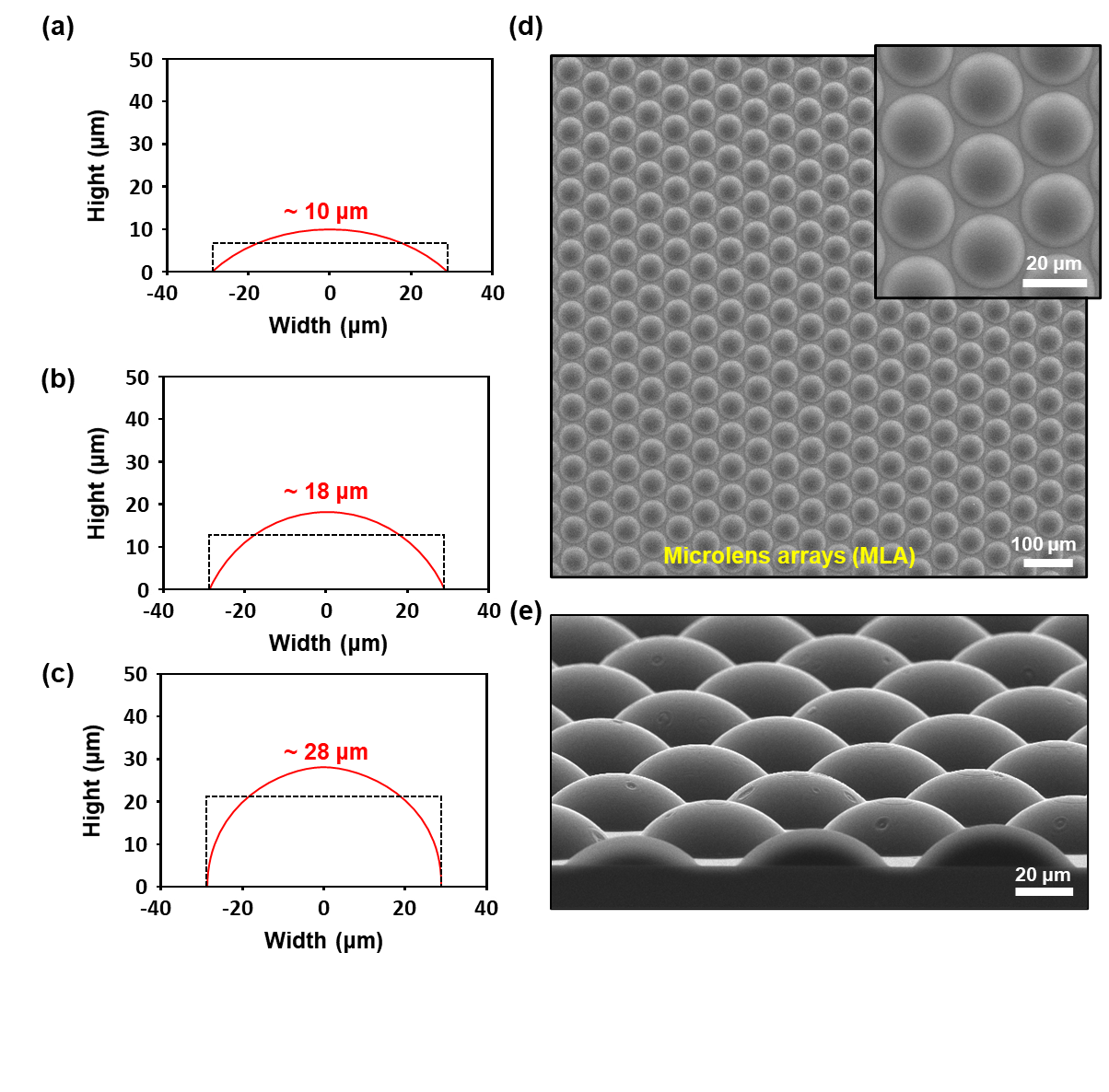


**Fig. S2** **a-c** The profiles of the microlens with different volume range (i.e., height to width). **d-e** Representative SEM images with close hexagonally packed MLA over a large-area after the reflow process with an optimized condition.


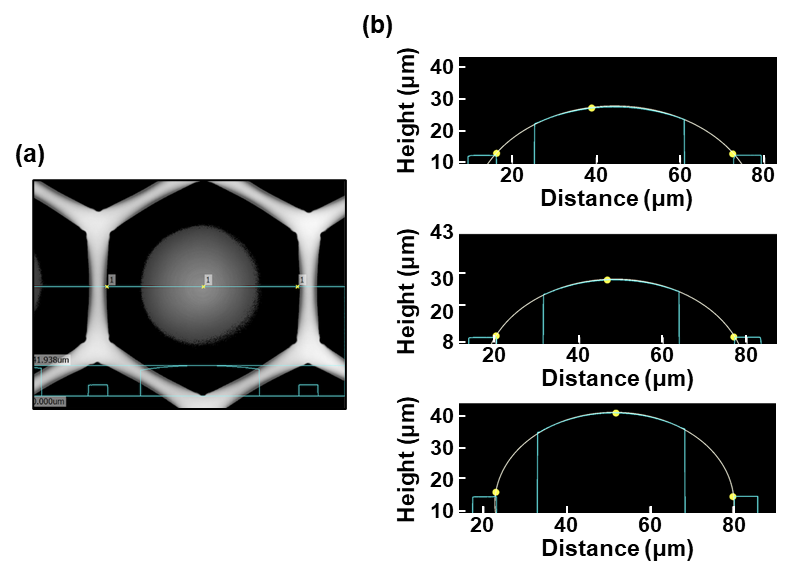


**Fig. S3** The height profiles of spherical shaped plano-convex MLA using 3D laser scanning microscope.


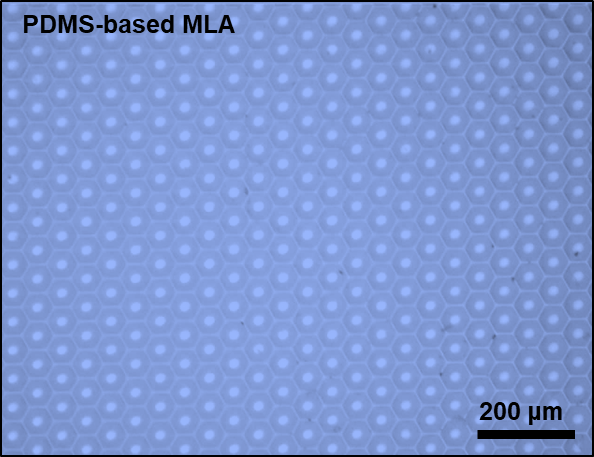


**Fig. S4** Optical micrograph of the replicated PDMS-based MLA over a large area from a Ni mold.


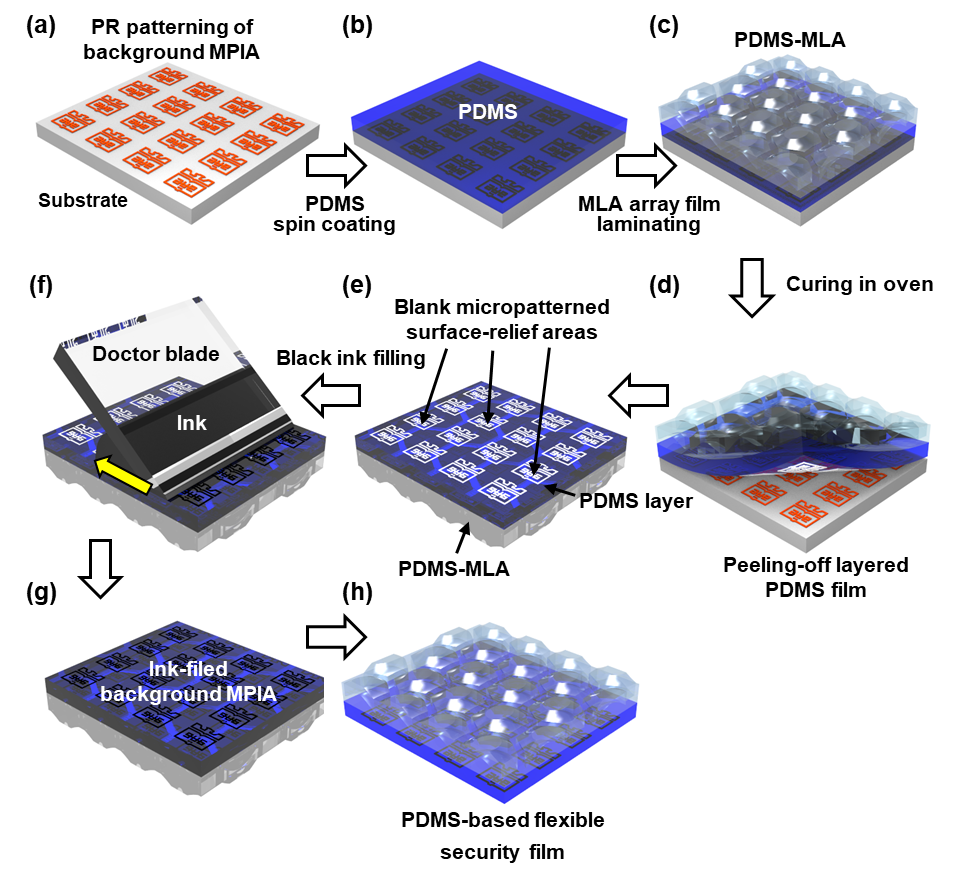


**Fig. S5** Sequentially description of schematics for fabricating flexible micro-image projection film. **a** Fabrication of logo-patterned arrays through a photolithography process. **b-d** Laminating of PDMS-MLA film and the peeling of the free-standing PDMS-casted MPIA film. **e-g** Black ink filling process on the MPIA regions. **h** Fabricated PDMS-based micro-image projection film as a security substrate.

**
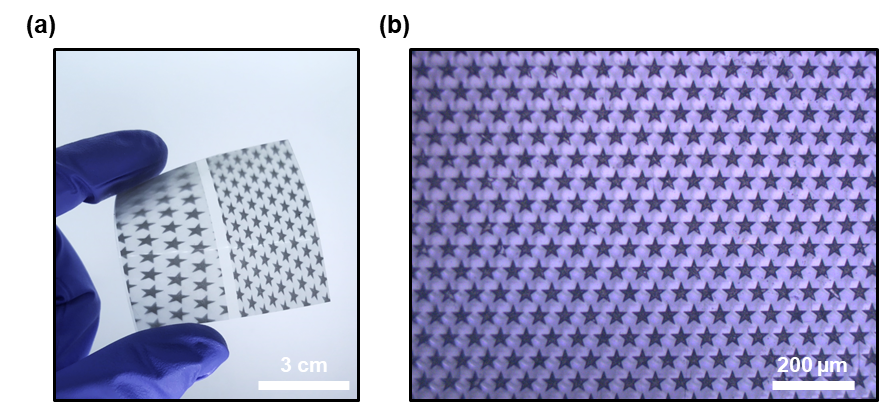
**

**Fig. S6** **a** Digital image of a elastomeric micro-image projected security substrate supported by PET carrier film. **b** Magnified optical micrograph of star-shaped PDMS MPIAs filled with black ink.

**
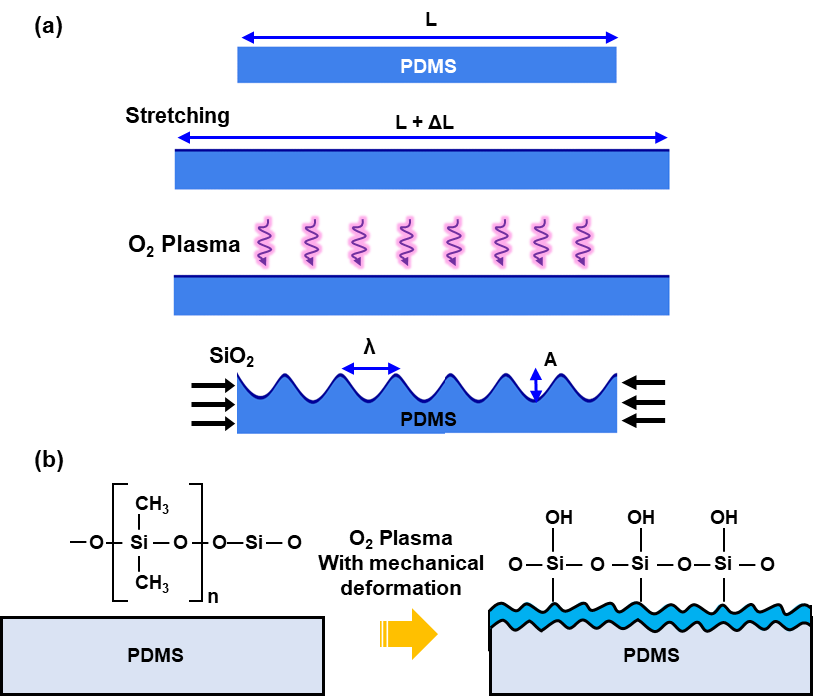
**

**Fig. S7** Principle of wrinkled nanostructure formation using O_2_ plasma treatment on PDMS substrate. **a** Schematic of sequential steps prestrain induced nanowrinkles on PDMS surface. **b** Chemical structure of PDMS and SiO_2_ layer formation by O_2_ plasma process and mechanical deformation.


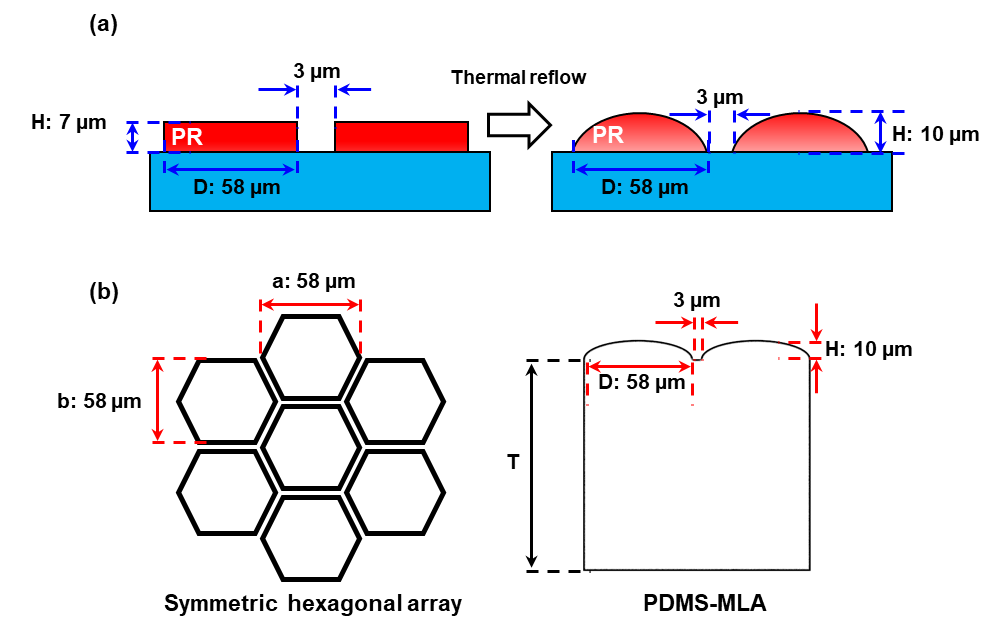


**Fig. S8 a** Hexagonally defined PR pillar-island can be transformed into hemispherical Plano-convex microlens by a thermal reflow process. **b** The dimensional information for the symmetrically ordered periodic arrays of microlenses (top and side views); the height of the MLA (H) is fixed, but the thickness of the MLA-sheet (T) can be controlled by the pressure onto the viscous PDMS prepolymer before the curing process.


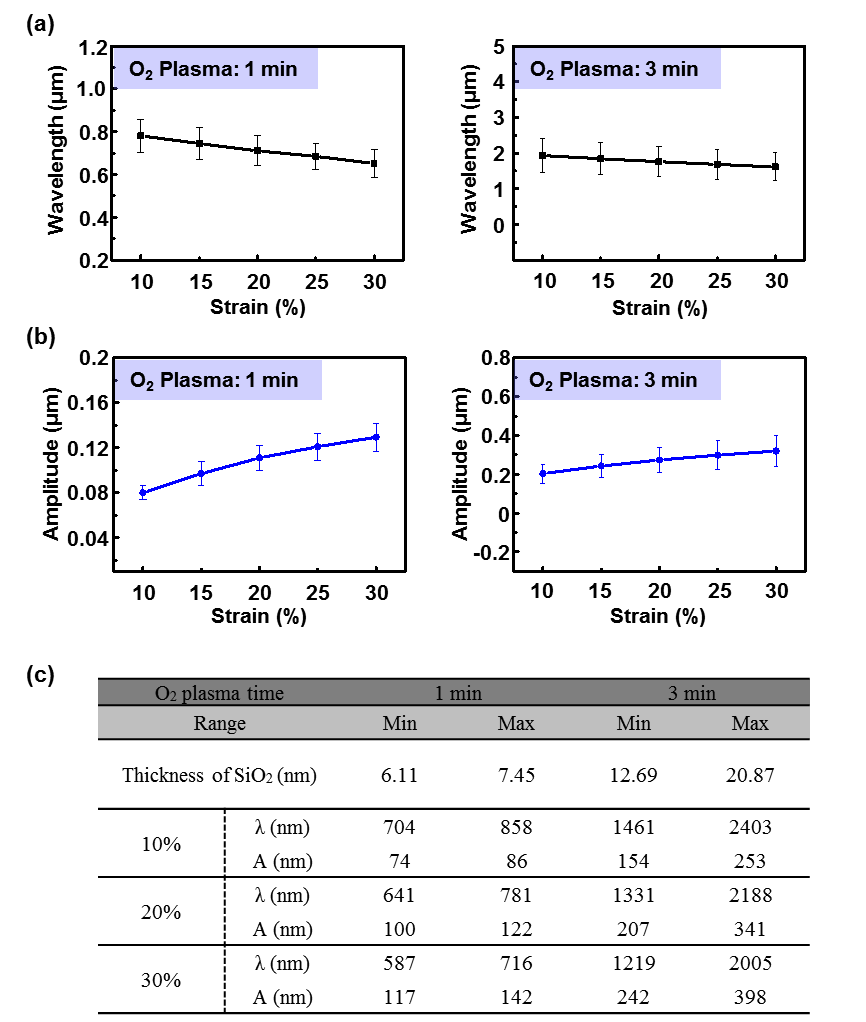


**Fig S9** Structural analysis using theoretical guidance on wrinkled nanostructures by O_2_ plasma treatment. **a-b** Wavelength and amplitude of nanowrinkles according to the processed strain ranges. **c** Table of the comprehensive set of results for various experimental conditions.

**Structural analysis of the periodic patterns of wrinkles and amplitude**

The formation of the periodic wrinkles is closely related to the mismatch in the mechanical properties of stiff thin film/elastic substrate (i.e., Young’s modulus, Poisson ratio, and film thickness). Nanostructured wrinkles are the result of minimizing total elastic energy when a stiff SiO_2_ layer formed on prestrained elastic PDMS substrate is subjected to large strains above a certain threshold. For small deformation ($\varepsilon<5\%$), wavelength ($\lambda_{0}$) of wrinkles can be expressed as follows:

$\lambda_{0}=2\pi h_{f}\left[\frac{E_{f}(1-v_{s}^{2})}{3E_{s}(1-v_{f}^{2})}\right]^{1/3}$ (1)

Here, $h_{f}$, *v*, and *E* are the thickness of thin film, Poisson ratio, and Young’s modulus, respectively; in the above equation, the subscripts of and *S* denote the film and substrate, respectively. $\overline{E}=E/(1-v^{2})$is the plain-strain modulus. This equation predicts that the wavelength of the wrinkles depends only on the thickness of the stiff thin film and plain-strain modulus ratio of film/substrate regardless of the prestrain factor ($\varepsilon_{pre}$). Hence, the amplitude ($A_{0}$) of wrinkling pattern is given as follows:

$A_{0}=h_{f}\sqrt{\frac{\varepsilon_{pre}}{\varepsilon}-1}$ (2)

The critical strain ($\varepsilon_{c}$) is defined as the minimum strain, which is a basic factor necessary for the wrinkles to occur (Eq. (3)).

$\varepsilon_{c}=\frac{1}{4}\left[\frac{3E_{s}(1-v_{f}^{2})}{E_{f}(1-v_{s}^{2})}\right]^{2/3}$ (3)

Based on the small-strain theory (Eq. (1) and (2)), the wavelength can be determined when the total strain is greater than critical strain ($\varepsilon_{c}$), which is independent of the applied tensile or compressive strain ($\varepsilon_{pre}$), while the amplitude increases as the applied strain increases.

However, for the large strain (i.e., $\varepsilon>5\%$), the elastomeric PDMS substrate becomes non-linear, and thus new model predicts the prestrain-dependent wavelength as follows:

$\lambda=\frac{\lambda_{0}}{(1+\varepsilon_{pre})(1+\xi)^{1/3}}$ (4)

Here, $\lambda_{0}$ is the wavelength based on the small-strain theory, and is the prestrain. The change of with arises from the geometrical non-linearity and a non-linear constitutive model for the substrate, including. When the compressive strain () is constant, the wavelength, can be determined by the film thickness. Also, the amplitude, *A* of wrinkles at large deformation can be predicted as follows:

$A=\frac{A_{0}}{\sqrt{1+\varepsilon_{pre}}(1+\xi)^{1/3}}$ (5)

where is amplitude based on small-strain theory. The Eq. (5) suggests that film thickness and prestrain coefficient can be used to extract wrinkle height. For example, in this study, we used = 40 GPa and = 0.3 for oxidized layer, and = 1.32 MPa and = 0.499 for the PDMS substrate.


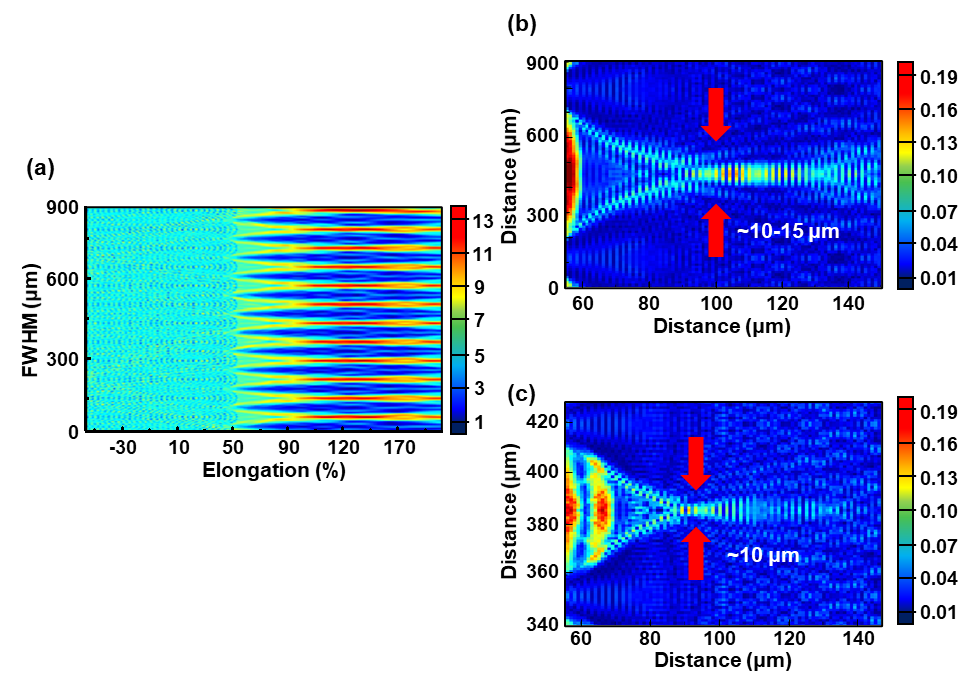


**Fig. S10 a** The electric field distribution of incident light through MLA optically simulated by the finite-difference time-domain (FDTD) method. a) the focal length of the MLA is defined as ~100 μm. **b-c** Focal length estimation by the propagation of a laser beam through a single lens; the thickness is set as 10 and 20 μm, respectively. By this experiment, the point spread function was found to be around ~10-15 μm.

**PDTD computed simulation**

We used a FDTD method to calculate electric field distribution for our fabricated MLA that is dimensionally arranged with 58 μm in diameter, 10 μm in height with refractive index, *n* of 1.428. The incident laser with 633nm wavelength was irradiated (normal incidence) on the PDMS-based MLA, focused around ~100 μm from the flat bottom surface of the MLA.


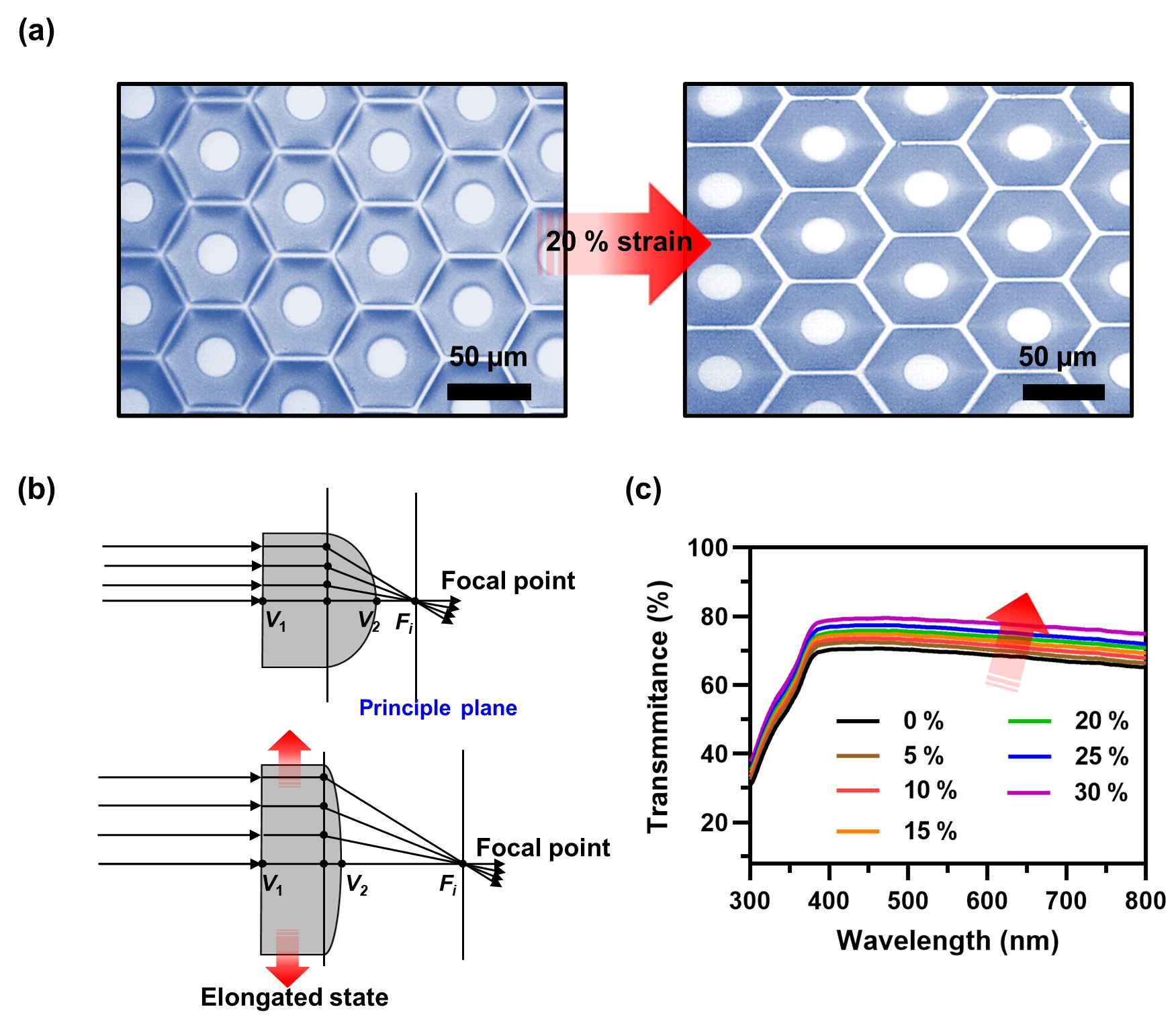


**Fig. S11 a** Optical micrographs on the initial and deformed state of flat-top MLA at the 20% strain; circular centered flat zone transforms to ellipsoidal shapes. **b** The schematic diagram of the focal point changes as the elastomeric MLA deforms to an elongated state. **c** The transmittance changes mesured by tuning the elastomeric MLA in a uniaxial direction.

**Focal length changes of PDMS-based MLA by the controlled deformation**

To obtain the focal length of the PDMS-based MLA at the deformed state, it's necessary to know the changes of height (i.e., hemispherical lens) from the initial state (10 μm) with the radius, *r* in 29 μm. Along with the deformation, we measured the changes in the radius of x- and y-axis (i.e., *R*_x_ and *R*_y_) using an optical microscope (Fig. S11a). For example, when the microlens was extended by 20%, the *R*_x_ was reduced from 29 to 26 μm, while the *R*_y_ was increased from 29 to 33 μm. Because the volume of the microlens was preserved during the elongation, the height of the microlens was naturally reduced from 10.5 to 9.8 μm when the microlens was elongated up to 20%. As shown in Figure S11b, the focal length of the elongated direction (*f*_y_) will be increased, and the focal length of the vertical direction (*f*_x_) will be reduced. Consequently, the *f*_x_ decreased from ~100 μm to ~92 μm, while the *f*_y_ increased from 100 μm to ~140 μm.


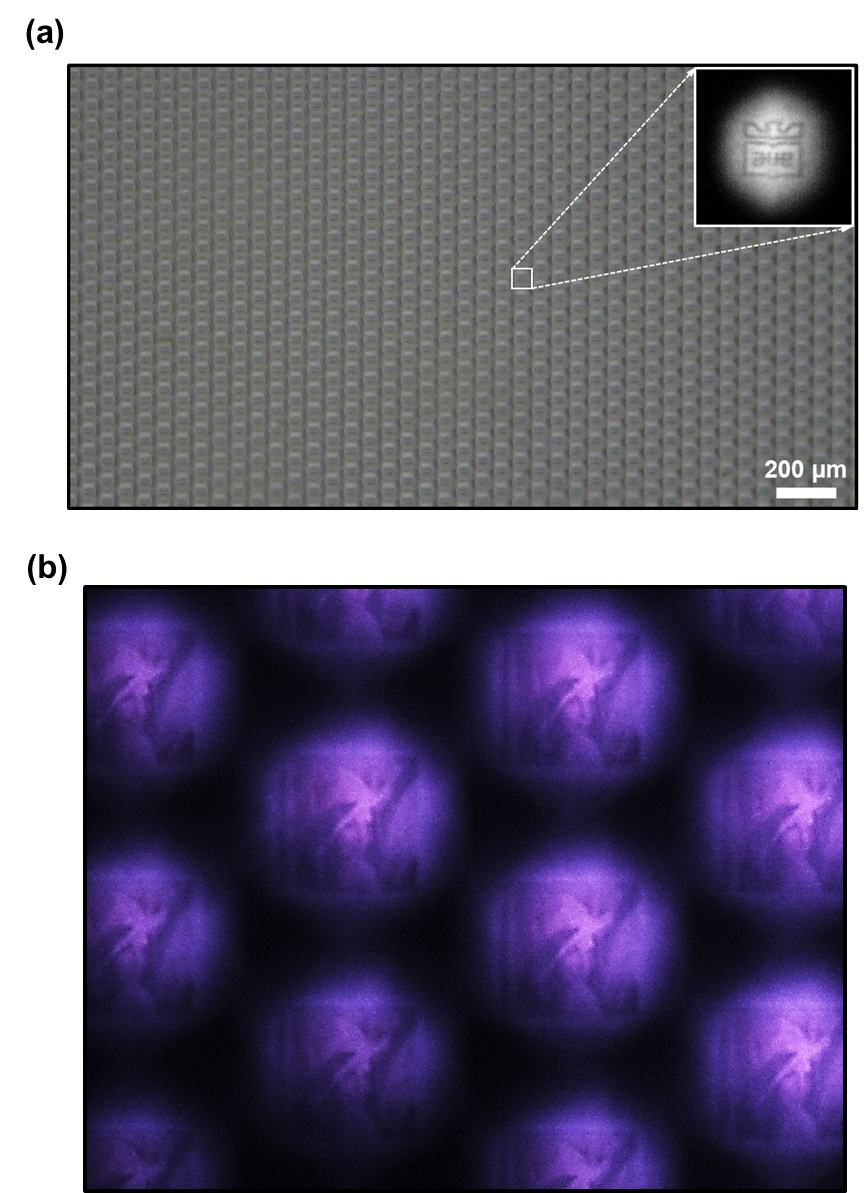


**Fig. S12** Optical micrographs as a result of the focused imaging by using a MLA for the university symbol, 20× magnification **a,** and Lenna image, 100× magnification **b**, located under the MLA (~50 mm).


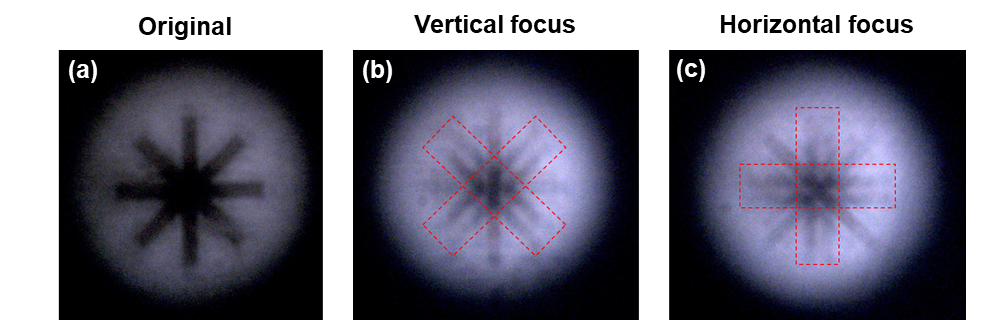


**Fig. S13** The virtual astigmatism test through the stretched MLA on a cross-bar image under an optical microscope. **a-c** The micrographs indicate unstretched original state, vertical focus with 5% elongation, and horizontal focus with 5% elongation, respectively.


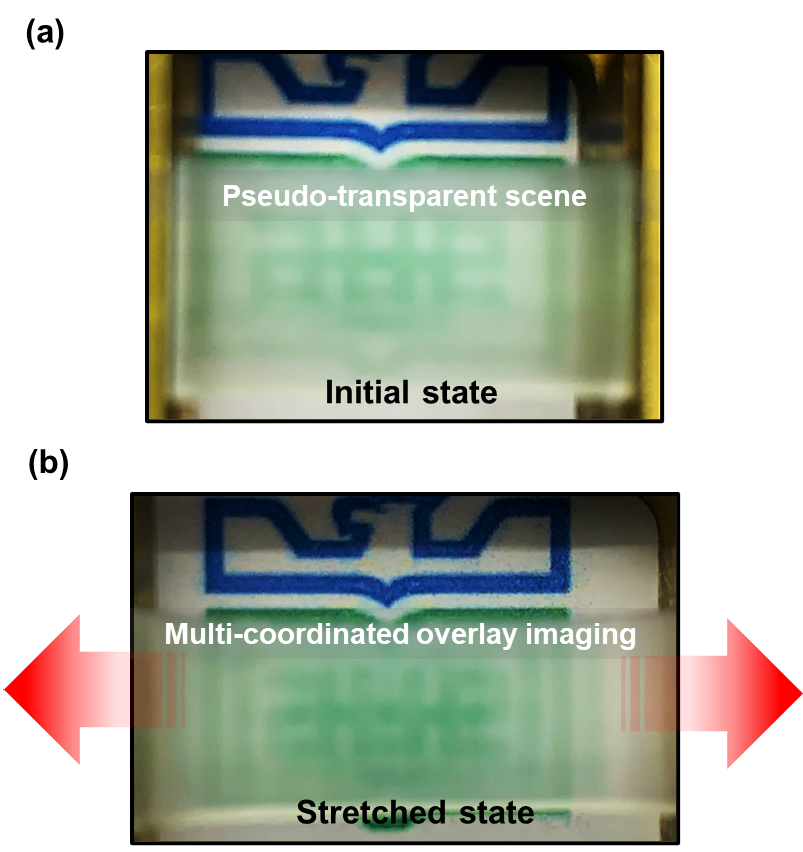


**Fig. S14** **a** Semi-transparent MLA fully structured with nanowrinkles. **b** Switchable multi-coordinated overlay image of a university symbol by stretching with biaxial direction.
